# Discrete choice analysis of health worker job preferences in Ethiopia: Separating attribute non‐attendance from taste heterogeneity

**DOI:** 10.1002/hec.4475

**Published:** 2022-02-17

**Authors:** Nikita Arora, Matthew Quaife, Kara Hanson, Mylene Lagarde, Dorka Woldesenbet, Abiy Seifu, Romain Crastes dit Sourd

**Affiliations:** ^1^ Faculty of Public Health and Policy London School of Hygiene and Tropical Medicine London UK; ^2^ Department of Health Policy London School of Economics and Political Science London UK; ^3^ School of Public Health Addis Ababa University Addis Ababa Ethiopia; ^4^ Centre for Decision Research, Management Division Leeds University Business School Leeds UK

**Keywords:** attribute non‐attendance, discrete choice experiment, health workers, preference heterogeneity

## Abstract

When measuring preferences, discrete choice experiments (DCEs) typically assume that respondents consider all available information before making decisions. However, many respondents often only consider a subset of the choice characteristics, a heuristic called attribute non‐attendance (ANA). Failure to account for ANA can bias DCE results, potentially leading to flawed policy recommendations. While conventional latent class logit models have most commonly been used to assess ANA in choices, these models are often not flexible enough to separate non‐attendance from respondents' low valuation of certain attributes, resulting in inflated rates of ANA. In this paper, we show that semi‐parametric mixtures of latent class models can be used to disentangle successfully inferred non‐attendance from respondent's “weaker” taste sensitivities for certain attributes. In a DCE on the job preferences of health workers in Ethiopia, we demonstrate that such models provide more reliable estimates of inferred non‐attendance than the alternative methods currently used. Moreover, since we find statistically significant variation in the rates of ANA exhibited by different health worker cadres, we highlight the need for well‐defined attributes in a DCE, to ensure that ANA does not result from a weak experimental design.

## 1 INTRODUCTION

Grounded in the economic theory of consumer behavior (Lancaster, 1966; McFadden, 1974), discrete choice experiments (DCEs) are popularly used by health economists for the valuation of health products and services (Soekhai et al., 2019). It is believed that DCE results can inform the allocation of healthcare resources, and support recommendations about welfare polices (de Bekker‐Grob et al., 2012; Lagarde et al., 2012; Mandeville et al., 2014; Rockers et al., 2012; Ryan, 2004; Saran et al., 2020).

Discrete choice experiments require respondents to process sizable amounts of information and it is typically assumed that respondents consider all available information before making their choices. However, a growing body of evidence now shows that respondents don't always behave this way, instead consciously or subconsciously use simple rules or heuristics to process information before making their decisions (Heidenreich et al., 2018; Hensher et al., 2005; Hess et al., 2013; Lagarde, 2013). One of these information processing strategies, attribute non‐attendance (ANA), relates to respondents only trading‐off a subset of the available attributes before choosing their preferred alternative. This violates the axiom of continuous preferences ‐ a key tenet of consumer theory and implies that respondents make trade‐offs between all attributes across each of the alternatives before making their decision (Campbell et al., 2008; Hensher et al., 2005; Hensher & Rose, 2009; Scarpa et al., 2009). Over the last decade, researchers have also increasingly acknowledged that failing to account for ANA may lead to biased coefficient estimates and a skewed understanding of respondent preferences (Heidenreich et al., 2018; Hole et al., 2013; Nguyen et al., 2015; Scarpa et al., 2009). However, accounting for ANA while assuming responder's choice to not consider all characteristics is always non‐attendance, when it could reflect preferences, can also result in the wrong cost‐benefit ratios and consequently distorted welfare estimates (Heidenreich et al., 2018).

In the DCE literature, a range of approaches have been used to account for ANA. These can broadly be classified into stated and inferred ANA. In stated ANA, analysts use respondent's self‐reported answers to indicate the extent to which they have ignored attributes (Collins, 2012; Hensher & Rose, 2009; Scarpa et al., 2009) while inferred ANA uses econometric modeling to estimate the probability with which respondents could have used different non‐attendance strategies (Campbell et al., 2008; Carlsson & Martinsson, 2003; Hensher et al., 2005; Hess et al., 2007; Hole et al., 2013; Lagarde, 2013). Both approaches restrict individual parameters of attributes that are considered to have been ignored, to zero. While the jury is still out about which is the most reliable method, the inference of ANA using an analytical approach has stronger appeal, especially when working with the understanding that non‐attendance in the dataset may partially reflect preferences. Previous studies have cautioned that respondnets' ability to reflect on their own decision making could be biased by their sub‐consicous preferences, questioning stated ANA methods to accurately capture non‐attendance (Heidenreich et al., 2018; Hensher & Rose, 2009; Hole et al., 2013). Although, econometric models used in inferring ANA can also produce results that are confounded with preference heterogeneity, if they are not flexible enough to separate respondent's genuinely low valuation of attributes, from ANA (Hensher et al., 2005; Hess et al., 2013; Hole et al., 2013). Our paper contributes to this literature by demonstrating the use of semi‐parametric models in the probabilistic determination of all possible ANA strategies used by a sample of frontline health workers in Ethiopia, while accounting for preference heterogeneity. We find that non‐attendance levels fall and model goodness‐of‐fit substantially improves when heterogeneity in respondent preferences is accounted for using discrete‐continuous latent class models (LCM). We also report that preferences for attributes and the extent of ANA varies with the cadre of health workers.

Not enough research has been done in health economics to assess if inferred ANA is a heuristic or genuine preference, especially using econometric models that are flexible enough to separate the two without relying on supplementary information from respondents. To our knowledge, one other study in the health context has used a similar econometric approach to ours where a mixed endogenous attribute attendance model was estimated to tease out preference heterogeneity from ANA using DCE data on the prescription behavior of Norwegian doctors (Hole et al., 2013). Ours will be the first application of this approach in a low‐and middle‐income country setting. Two factors underlie the importance of study context, and the value of applying an improved approach to the econometric inference of ANA in LMICs. First, there is some literature that suggests that ANA maybe a greater threat to the validity of DCE results in LMICs, than in higher‐income settings. Nguyen et al. (2015) reviewed relevant DCEs conducted in developed and developing counties and used their results on ANA from a DCE conducted in Vietnam to demonstrate that rates of ANA are on average higher in developing countries than in developed ones. Second, the application of advanced econometric modeling techniques to identify ANA in health workers' employment preferences in Ethiopia is important because ANA potentially undermines the validity of marginal valuations. Generating valid estimates is important if research is to inform policy.

## 2 DATA

We used data from a DCE designed to quantitatively assess the job preferences of health workers based in four regions in Ethiopia: Tigray, Amhara, Oromia and Southern Nations, Nationalities, and People's Region, which altogether make up for more than 80% of the country's total population. Many DCEs have been conducted to understand the job incentives that align best with the preferences of doctors and nurses in LMICs (Mandeville et al., 2014, 2016, 2017; Smitz et al., 2016; Song et al., 2015), which is relevant in improving their retention in the workforce. However, only limited quantitative research is available on the job preferences of medium and low‐skilled health workers like midwives and community health workers, who are often the backbone of primary healthcare delivery in countries like Ethiopia. In our study, we focus on understanding the job preferences of three frontline health worker cadres: community health workers called *health extension workers (HEW); mid‐level healthcare providers* including midwives; and *non‐patient facing staff* such as health facility administrators. More details about the three cadres and the health labor market in Ethiopia can be found in our previously published work with these health workers in Lamba et al. (2021). This DCE was embedded within a survey collecting endline information for a process evaluation of a quality improvement (QI) program targeted to improve the knowledge and motivation of these health worker cadres, implemented by the Ministry of Health in Ethiopia. The study found that the QI program had almost no impact on health worker motivation, but some impact on health worker knowledge. The DCE was added to this data collection as a standalone module to investigate job preferences of different cadres (Lamba et al., 2021).

The endline survey was conducted in June 2019, with a cadre‐stratified random sample of 404 health workers in the Ethiopian health system, where 68% (275) of the original sample was re‐interviewed along with 129 newly recruited respondents. The sample comprised 202 HEWs (50%); 40 non‐patient facing staff (10%); and 162 mid‐level healthcare providers (40%). A target sample size of 50 respondents per region was chosen, based on the primary research question of assessing changes in motivation as measured by Likert scale questions. After piloting, the largest S‐estimate for any level of the final design was checked for consistency with this sample size – this was 184, so the design was deemed to give a good chance of giving significant parameters at the 5% level. Since one of our key findings in Lamba et al. (2021) was that health worker preferences differed by their cadre type, we hypothesized that cadre will also impact the rates of ANA for different job attributes. To make the estimation of these complex models computationally possible in a reasonable time frame, we split the dataset into two and present results among HEWs and other cadres separately.

A team of seven trained research assistants from Addis Ababa University administered the DCE, face‐to‐face with the respondents, using Tigringya, Amharic, and Oromifa languages and Open Data Kit (https://opendatakit.org) software on tablet computers. To reduce social desirability bias in responses, we allowed research assistants to explain the experiment to respondents, after which they were told to make a decision about their preferred job on their own.

Informed consent was obtained from all participants before data were collected, and the study was undertaken with ethical approval from the Observational Research Ethics Committee of the London School of Hygiene and Tropical Medicine and a program evaluation waiver from the Ethics Committee of the Ethiopian Public Health Association.

### 2.1 Discrete choice experiment development and design

The DCE had six attributes, identified after a thorough review of literature on health workforce choice experiments conducted in the East African context (Blaauw et al., 2013; Mandeville et al., 2017; Mangham & Hanson, 2008; Rockers et al., 2012). Ten potential attributes were initially shortlisted and eventually reduced to six, guided by the findings of a qualitative study conducted in Ethiopia, a year previously to data collection (Wang et al., 2016). The selected attributes described pecuniary and non‐pecuniary workplace incentives, facility and management structures characterizing the key features of the jobs of all three sampled cadres. Table 1 provides the final list of attributes included in the DCE along with their levels; each attribute level was dummy coded as 0 or 1. From these attributes, 216 (3^3^ × 2^3^) possible combinations of jobs could have been formed.

**TABLE 1:** DCE attributes and their levels

Attributes	Attribute levels
Salary	20% below average
	Average earnings
	20% above average
Training	No training available
	5 days per year dedicated training time (improving work‐related and transferable skills)
	10 days per year dedicated training time (improving work‐related and transferable skills)
Workload	Light: More than enough time to complete duties
	Medium: Enough time to complete duties
	Heavy: Barely enough time to complete duties
Management style	Management is supportive, and makes work easier
	Management is not supportive, and makes work more difficult
Health facility quality	Your workplace is good: It has reliable electricity and other services, supplies are always available
	Your workplace is basic: It has unreliable electricity, whilst supplies you need are not always available
Opportunities to improve health outcomes	Your work will have a large impact on improving health in the local community
	Your work will have a small impact on improving health in the local community

Abbreviation: DCE, discrete choice experiments.

The DCE was piloted among 19 district health office staff in December 2017, before the baseline survey for the main study was conducted. The pilot had a ten‐task fractional factorial design while the final was a seven‐task, D‐optimal design based on priors from the pilot, conducted in NGENE (Choice Metrics, 2012). The design was main effects only, and because it was a subsection of a survey which took a relatively long time to complete, no additional quality check tasks were included for example, dominance checks or repeat tasks**.**

**FIGURE 1:**
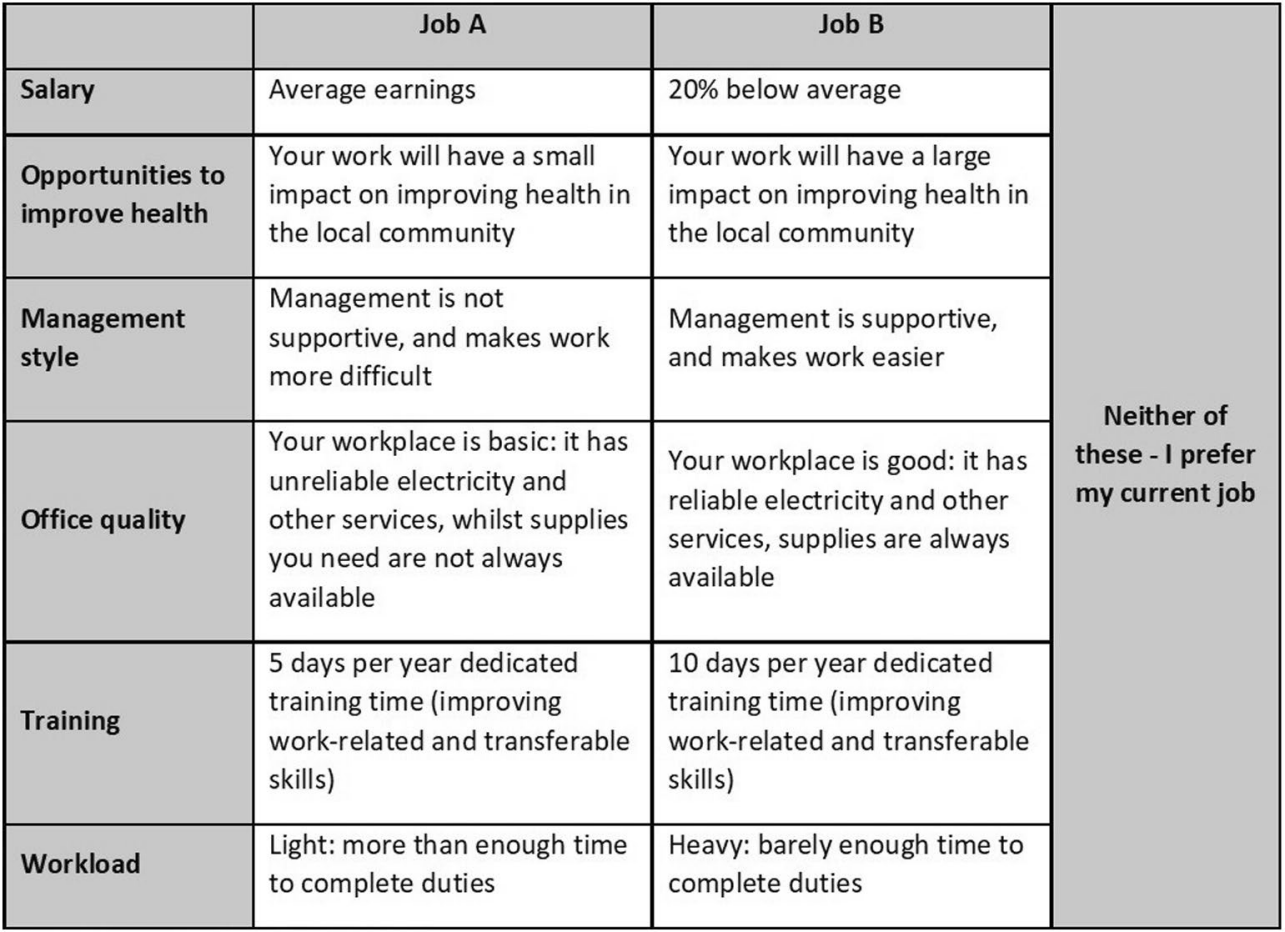
Example choice task

Each task displayed two, unlabeled job alternatives described by six attributes, where each alternative represented a generic health worker's job in Ethiopia. Participants were asked the following question: “*Here are two jobs described by some of their characteristics. Compared to your current job, please choose which you would prefer”.* Respondents were also explained that barring the given attributes, all other characteristics in the jobs were identical. Figure 1 shows an example choice task, as presented to the respondents.

To increase realism and allow for the estimation of unconditional demand, a generic opt‐out alternative, modeled simply as a constant with no attribute levels, was included to represent the choice of picking neither of the presented job profiles and staying in their current jobs. We used Apollo version 0.2.4 (Hess & Palma, 2019) in R (version 4.0.2) to analyze our data.

## 3 METHODOLOGICAL APPROACH

### 3.1 The mixed multinomial logit model

Standard random utility models in DCEs are based on the framework by McFadden (1974), and believe that respondent choice is determined by the utilities that they perceive for given alternatives. For respondent n, alternative i, and choice situation t, utility, U, can be given by

(1) $$U_{i,n,t}=V_{i,n,t}+\epsilon_{i,n,t}$$

where, U, is made up of a deterministic component $V_{i,n,t}$, and a random component $\epsilon_{i,n,t}$ which is assumed to be an independently and identically distributed Extreme Value Type I function (Hensher et al., 2005; Manski, 2001). Further, the deterministic part of the utility can be re‐written as:

(2) $$V_{i,n,t}=f\left(\beta_{n},x_{i,n,t},z_{n}\right)$$

where $\beta_{n}$ is the vector of sensitivities for the respondent, $x_{i,n,t}$ is a vector of attributes for alternative i and $z_{n}$ is a vector of socio‐demographic characteristics of respondent n.

In this DCE application, the deterministic utility for a job alternative i, for individual n, characterized by a selected set of six attributes, can be given by:

(3) $$\begin{array}{lll} \beta_{n}x_{n,i,t} & = & \beta_{asci}+\beta_{1}Salary_{i}+\beta_{2}Impact_{i}+\beta_{3}Management_{i}+\beta_{4}Facility_{i}\\ & & +\beta_{5}Training_{i}+\beta_{6}Workload_{i} \end{array}$$

where $\beta_{asci}$ corresponds to an alternative‐specific constant for alternative i. Two ASCs were featured in the models given that there were three alternatives in each choice task. $\beta_{1}$ to $\beta_{6}\;$ represent the preference weights of attributes used to characterize job alternatives included in the DCE.

Typically, a multinomial logit model (MNL) is used to estimate the probability with which each respondent makes a sequence of choices. However, since the model is restrictive and assumes all respondents to have the same preferences for a given attribute, we start our estimation with a mixed multinomial logit model (MMNL) which allows us to relax this assumption and for the coefficients to follow a distribution.

If $f\left(\theta_{n}|\Omega \right)$ is the joint density over taste parameters, where $\theta_{n}$ is a vector of random parameters and Ω the parameter of the distributions, using an MMNL the probability of the sequence of choices, $Y_{n},$ made by respondent *n* can be given by:

(4) $$\mathrm{Pr}\left(Y_{n}|x_{n},\Omega \right)=\int \underset{t\in T_{n}}{\prod }\frac{\exp\left(\beta^{\prime }x_{nit}\right)}{\sum_{j\in J}\beta^{\prime }x_{njt}}f\left(\theta_{n}|\Omega \right)d\left(\theta_{n}\right)$$

Even though the MMNL does not capture ANA in the dataset, we estimate it as a base model to gradually build up model complexity as well as to include it in the comparison of goodness of fit between different models.

### 3.2 Latent class model for attribute non‐attendance

Using discrete distributions to model the underlying preferences of respondents, LCMs are popular semi‐parametric specifications that accommodate response heterogeneity in choice models (Hensher et al., 2005). LCMs assume that the behavior of respondents depends on observable attributes and on latent heterogeneity that varies with factors observed by the analyst. In an LCM, the population of respondents can be divided into a set number of *Q* classes that differ in their preferences. While preferences are assumed to be different between classes, within each class all members are assumed to share the same preferences (Hensher et al., 2005). The model assumes that class allocation is probabilistic and which class contains any particular individual is unknown to the analyst. In a conventional LCM that is modeling heterogeneity in preferences, the optimal number of classes to be included is normally determined by noticing the change in model goodness of fit as the number of classes go up one‐by‐one. This can be done by monitoring an information criterion like Akaike Information Criterion (AIC) or Bayesian Information Criterion (BIC) which penalizes model fit as the number of parameters increase (Heidenreich et al., 2018). In contrast, a latent class model for attribute non‐attendance (ANA‐LC) estimates a behavioral model which assumes that respondents use heuristics in processing information in a DCE, and only attend to a subset of the given K attributes. This results in $2^{K}$ different combinations of ANA and each combination can be given by a class in the ANA‐LC (Collins, 2012; Hensher et al., 2005; Lagarde, 2013). With six attributes in our sample, we estimated sixty four (2^6^) latent classes in our ANA‐LC. Estimating an LCM with 64 classes using the standard practice of estimating a constant for each of the 64 classes (minus one), could have proved to be burdensome and reduced model parsimony substantially due to a spike in the number of estimated parameters. So, following the approach by Hole et al. (2013), we estimated a constant for each of the six attributes instead, and generated the probability of an attribute being attended to (or not) over all 64 combinations, by introducing a binary logit model for each of the attributes. This increased the number of estimated parameters in the model by six, not 63. A drawback of the specification, however, is that it is important to assume that the non‐attendance probabilities are independent. A detailed description of this specification is provided in the Supporting Information S1 accompanying this article, but as an illustration, we show that the probability ω that all the attributes were attended to, corresponds to:

(5) $$\omega_{Complete\;attendance}=\frac{\exp\left(\delta_{salary}\right)}{1+\exp\left(\delta_{salary}\right)}\cdot \frac{\exp\left(\delta_{training}\right)}{1+\exp\left(\delta_{training}\right)}\cdot \frac{\exp\left(\delta_{workload}\right)}{1+\exp\left(\delta_{workload}\right)}\cdot \frac{\exp\left(\delta_{quality}\right)}{1+\exp\left(\delta_{quality}\right)}\cdot \frac{\exp\left(\delta_{management}\right)}{1+\exp\left(\delta_{management}\right)}\cdot \frac{\text{exp}\left(\delta_{opportunities}\right)}{1+\text{exp}\left(\delta_{opportunities}\right)}$$

While the probability of a combination where all attributes were attended to except for *salary* and *workload,* corresponds to:

(6) $$\omega_{salary\;and\;workload\;non‐attendance}=\frac{1}{1+\exp\left(\delta_{salary}\right)}\cdot \frac{\exp\left(\delta_{training}\right)}{1+\exp\left(\delta_{training}\right)}\cdot \frac{1}{1+\exp\left(\delta_{Workload}\right)}\cdot \frac{\exp\left(\delta_{quality}\right)}{1+\exp\left(\delta_{quality}\right)}\cdot \frac{\exp\left(\delta_{management}\right)}{1+\exp\left(\delta_{management}\right)}\cdot \frac{\text{exp}\left(\delta_{opportunities}\right)}{1+\text{exp}\left(\delta_{opportunities}\right)}$$

Equations (5) and (6) are adaptions of the equations used for similar analysis by Hole et al. (2013). The extent to which a single attribute, say salary, was ignored could also be calculated by simply imputing the value of $\delta_{salary}$, calculated using the ANA‐LC, in the salary component of Equation (6).

### 3.3 Assessing patterns of ANA using discrete‐continuous mixture models

In health economics literature, LCMs that simply account for all or a reduced version of the possible 2^k^ strategies have been considered to be sufficient for estimating the patterns of ANA in a dataset (Heidenreich et al., 2018; Lagarde, 2013). However, if substantial preference heterogeneity unrelated to ANA exists, such LCMs are likely to give results that are confounded by respondent's taste heterogeneity (Hess et al., 2013; Hole et al., 2013). As a result, the share of respondents that get allocated to a non‐attendance class don't necessarily have zero sensitivity toward the attribute but a relatively low sensitivity, and that real non‐attendance is rarer than imagined thereby generating misleading model estimates (Campbell et al., 2008; Collins, 2012; Hess et al., 2013).

In order to distinguish preference heterogeneity from ANA in our dataset, we estimated a logit model that combined discrete and random parameters (Hess et al., 2013; Hole et al., 2013). The resultant model, which we called “ANA‐MMNL”, accounted for continuous taste heterogeneity in respondent preferences while inferring all 64 permutations of ANA. The probability of observing a sequence of choices made by a given respondent *n* according to the ANA‐MMNL model, thus correspondeds to:

(7) $$\mathrm{Pr}\left(Y_{n}|x_{n},\Omega \right)=\sum_{q\in Q}\omega_{nq}\int \prod_{t\in T_{n}}\frac{\exp\left(\beta^{\prime }x_{nit}\right)}{\sum_{j\in J}\beta^{\prime }x_{njt}}f\left(\theta_{n}|\Omega \right)d\left(\theta_{n}\right)$$

We compare ANA models without and with mixing in the paper, so the within‐class probabilities of the former corresponds to MNL, not MMNL. All MMNL and ANA‐MMNL models, which accounted for random heterogeneity in respondent preferences, were estimated using 5000 Sobol draws where all attribute levels followed a normal distribution except higher than average salary, which we constrained to positive lognormal based on the expectation that all respondents will gain utility from this level. During the initial estimation of some ANA‐MMNL models we found that certain attributes were always attended to, resulting in very large values of their delta parameters. In such cases, we re‐estimated the final models after excluding the corresponding ANA classes for these parameters, to ensure model parsimony and convergence (Hess et al., 2013).

## 4 RESULTS

We start by presenting our goodness of fit results, followed by estimation results from the models that perform best, for each of the two sub‐samples. Finally, we compare the rates of ANA between ANA‐LC and ANA‐MMNL models, disaggregated by cadre type.

### 4.1 Model fit

Table 2 reports the BIC, AIC and log‐likelihood of the three main models ‐ MMNL, ANA‐LC and ANA‐MMNL for both sub‐samples. For the dataset with HEWs, we see that the ANA‐MMNL outperforms the other models on all three measures of fit. This was expected as the ANA‐MMNL provides gains in efficiency by allowing further flexibility in the distribution of preferences across respondents, while maintaining model parsimony by including only 6 additional parameters to the model. For Other cadres, we see that while ANA‐MMNL outperforms the other models on AIC and log‐likelihood, it gets penalized for the number of parameters by the BIC where MMNL outperforms it. This was not surprising as the penalty term for the number of parameters included in the model is larger in BIC than in AIC, and we believed that all parameters entering the model at this stage were necessary for successfully inferring ANA.

**TABLE 2:** Goodness of fit results

		MMNL	ANA‐LC	ANA‐MMNL
HEW	AIC	2397.13	2531.22	2386.16
	BIC	2512.7	2620.53	2506.99
	Log‐likelihood	−1176.56	−1248.61	−1170.08
Other cadres	AIC	2555.01	2697.31	2550.21
	BIC	2670.60	2818.15	2707.83
	Log‐likelihood	−1255.51	−1325.65	−1245.10

Abbreviations: AIC, Akaike information criterion; ANA‐LC, latent class model for attribute non‐attendance; ANA‐MMNL, discrete‐continuous mixtur; BIC, Bayesian information criterion; HEW, health extension workers; MMNL, mixed multinomial logit model.

To confirm our results and to assess if the ANA‐MMNL statistically supersedes the other models, we present results from Likelihood Ratio tests between MMNL and ANA‐MMNL; and ANA‐LC and ANA‐MMNL in Table 3. These results were consistent with our expectations. We show strong statistical evidence in favor of ANA‐MMNL outperforming the other models for both the sub‐samples.

**TABLE 3:** Likelihood Ratio test results: ANA‐MMNL outperforms ANA‐LC and MMNL

Models	Parameters	Models	Parameters
HEW			
ANA‐LC	17	MMNL	22
ANA‐MMNL	23	ANA‐MMNL	23
Difference	6	Difference	1
LR test *p*‐value	<0.001	LR test *p*‐value	<0.001
Other cadres			
ANA‐LC	23	MMNL	22
ANA‐MMNL	30	ANA‐MMNL	30
Difference	7	Difference	8
LR test *p*‐value	<0.001	LR test *p*‐value	0.008

*Note*: The MMNL and ANA‐LC are restricted versions of the ANA‐MMNL. ANA‐MMNL is the urestricted model in these Likelihood ratio tests.

Abbreviations: ANA‐LC, latent class model for attribute non‐attendance; ANA‐MMNL, Discrete‐continuous mixture mode; HEW, health extension workers; LR, likelihood ratio; MMNL, mixed multinomial logit model.

### 4.2 Estimation results

Since the ANA‐MMNL models fitted our data best for both the sub‐samples, below we only present results from these models. Class membership for non‐attendance was calculated using Equation (5), which provided estimates for the extent of non‐attendance (*δ*) of each attribute. As the values of *δ* parameters decreased, ANA increased. Rates of non‐attendance are presented in Table 6 and discussed in detail in the following section. Table 4 gives ANA‐MMNL results for HEWs. We report that HEWs preferred good management, lower number of training days, and good facility quality. They showed disutility toward a heavy workload, higher number of training days and average salary. Its worth noting that while the mean preferences of HEWs for *medium workload* were insignificant, there was statistically significant heterogeneity in the sample for preferences toward that attribute level.

**TABLE 4:** Estimation results of ANA‐MMNL, for HEWs

No. of observations	1413		
No. of respondents	202		
McFadden's pseudo *R* ^2^	0.2462		
Category	Parameter	Coefficient	Robust T ratio
Attribute mean (*μ*)	*Asc for job 1*	−0.090	−1.32
	*Asc for opt out*	−3.427^***^	−4.70
	*Avg. Salary*	−0.434^***^	−3.30
	*20% more than avg. salary*	0.022^a^	−1.15
	*5 days training*	0.556^**^	2.49
	*10 days training*	−0.835^**^	−2.79
	*Medium workload*	3.037	0.64
	*Heavy workload*	−1.922^**^	−2.45
	*Good facility quality*	0.260^**^	2.31
	*Good management*	0.929^***^	5.50
	*Good opportunities to improve health*	−0.105	−0.39
Attribute standard deviation (*σ*)	*Asc for job 1*	0.032	0.36
	*Asc for opt out*	2.985^***^	7.07
	*Avg. Salary*	−0.003	−0.33
	*20% more than avg. salary*	2.304	1.47
	*5 days training*	−0.649^*^	−1.92
	*10 days training*	0.490	1.10
	*Medium workload*	−4.540^**^	−2.26
	*Heavy workload*	−0.579	−0.50
	*Good facility quality*	−0.819^***^	−5.50
	*Good management*	−0.005	−0.31
	*Good opportunities to improve health*	−0.423	−0.75
Extent of non‐attendance (*δ*) for HEWs	*Workload*	−0.775	−0.77

*Note*: As stated above, in our estimation of the ANA‐MMNL for HEWs, all attributes except *Workload* were always attended to (had 0% non‐attendance). They were thus excluded from final model estimation. The opt‐out was selected 11.5% of the times.

^a^ Since more than average salary had a positive log normal distribution, the coefficient presented in Table 4 is the exponent of the actual value: −3.822.

^***^Significant at 1% level, ^**^significant at 5% level, ^*^significant at 10% level.

Further, Table 5 gives the mean preferences for the pooled sample comprising Other cadres. We find that respondents from Other cadres preferred a medium workload, good facility quality, good management and a higher than average salary. They disliked a higher number of training days and receiving an average salary. They were also more likely to choose to stay in their current job, that is, choose the opt‐out rather than either of the two hypothetical jobs.

**TABLE 5:** Estimation results of ANA‐MMNL, for Other cadres

No. of observations	1414		
No. of respondents	202		
McFadden's pseudo *R* ^2^	0.1985		
Category	Parameter	Coefficient	Robust T ratio
Attribute mean (*μ*)	*Asc for job 1*	−0.142^**^	−2.45
	*Asc for opt out*	−2.336^***^	−6.24
	*Avg. Salary*	−0.597^***^	−3.92
	*20% more than avg. salary*	0.171^a^ ^,^^**^	−1.87
	*5 days training*	0.222	1.02
	*10 days training*	−0.889^***^	−4.19
	*Medium workload*	2.710^***^	3.10
	*Heavy workload*	−3.344	−0.88
	*Good facility quality*	0.210^**^	2.04
	*Good management*	0.574^***^	3.96
	*Good opportunities to improve health*	0.244	1.40
Attribute standard deviation (*σ*)	*Asc for job 1*	0.001	0.38
	*Asc for opt out*	2.322^***^	8.08
	*Avg. Salary*	0.001	0.10
	*20% more than avg. salary*	1.257^**^	2.42
	*5 days training*	−0.670^**^	−2.93
	*10 days training*	−0.444	−1.08
	*Medium workload*	0.052	0.53
	*Heavy workload*	2.066	1.19
	*Good facility quality*	−0.439^**^	−2.29
	*Good management*	−0.001	−0.15
	*Good opportunities to improve health*	−0.004	−0.21
Extent of non‐attendance (*δ*) for mid‐level healthcare providers	*Salary*	10.977	0.94
	*Training*	14.630^***^	8.29
	*Workload*	−2.030^**^	−2.76
	*Opportunities to improve health*	9.125^***^	5.66
Extent of non‐attendance (*δ*) for non‐patient facing staff	*Salary*	−0.552	−0.29
	*Training*	1.689	0.49
	*Workload*	−0.880^*^	−1.71
	*Opportunities to improve health*	−14.754^***^	−7.42

*Note*: As stated above, in our estimation of the ANA‐MMNL for Other cadres, workload and management were always attended to. They were thus excluded from final model estimation. The opt‐out was selected 11.5% of the times.

^a^ Since more than average salary had a positive log normal distribution, the coefficient presented is the exponent of the actual value, −1.765.

^***^Significant at 1% level, ^**^significant at 5% level, ^*^significant at 10% level.

### 4.3 Rates of ANA across models

Table 6 gives the rates of ANA across ANA‐LC and ANA‐MMNL models for all three cadres. Starting with rates of non‐attendance for ANA‐LC models, we see that the most ignored attribute by HEWs was *salary*, followed by *workload*. There was substantial non‐attendance for *opportunities to improve health outcomes* and *facility quality* with over 70% of HEWs ignoring them*. Training* and *management* were the only attributes where non‐attendance was less than 50%. For the same model, we see that the rates of ANA exhibited by Other cadres were quite different from HEWs but similar between mid‐level healthcare providers and non‐patient facing staff. Mid‐level healthcare providers show very high rates of ANA for all attributes except *opportunities to improve health outcomes,* similarly to non‐patient facing staff with the only difference that non‐patient facing staff attend to salary a lot more than any other cadres with only 35% not attending to it.

**TABLE 6:** Rates of ANA captured in different ANA models

*Attribute*	ANA‐LC						ANA‐MMNL					
	*HEW*	*T ‐ratio*	*Other cadres*				*HEW*	*T‐ ratio*	*Other cadres*			
			*Non‐patient facing*	*T ‐ratio*	*Mid‐level provider*	*T‐ratio*			*Non‐patient facing*	*T ‐ratio*	*Mid‐level provider*	*T‐ ratio*
Salary	100%	>10^**^	91%	2.2^*^	35%	0.4	0%	‐	63%	1.5	0%	0.0
Training	48%	8.8^**^	95%	7.7^**^	84%	5.7^*^	0%	‐	16%	0.4	0%	0.6
Workload	83%	>10^**^	71%	3.3^**^	74%	0.4	68%	3.2^**^	71%	6.6^**^	88%	>10^**^
Facility quality	70%	>10^**^	80%	3.8^**^	82%	0.8	0%	‐	0%	‐	0%	‐
Management	22%	1.9^*^	99%	0.0	67%	0.0	0%	‐	0%	‐	0%	‐
Health outcomes	72%	8.1^**^	34%	0.1	40%	0.2	0%	‐	100%	>10^**^	0%	0.6

*Note*: Standard errors and robust T ratios were estimated using the Delta method (Oehlert, 1992).

Abbreviations: ANA‐LC, latent class model for attribute non‐attendance; ANA‐MMNL, Discretecontinuous mixture mode; HEW, health extension workers.

^*^Significant at the 5% level, ^**^significant at the 1% level.

On the contrary to the above, we note that ANA‐MMNL models report drastically lower rates of ANA in comparison to ANA‐LC models, in line with our hypothesis that these models allow respondents' low preferences to be separated from non‐attendance. HEWs seem to completely attend to all attributes except workload, similarly to mid‐level healthcare providers, while non‐patient facing staff show complete attendance only for *management* and *opportunities to improve health outcomes.* This cadre shows complete and substantial non‐attendance for *facility quality* and *workload,* respectively, while lower rates for *salary* and *training*.

## 5 DISCUSSION AND CONCLUSIONS

Overall, our findings support the growing evidence that a significant proportion of participants ignore attributes in choice experiments. There are still only a few studies that have accounted for ANA in the health economics literature, though this number is slowly increasing (Erdem et al., 2015; Heidenreich et al., 2018; Hole et al., 2013; Lagarde, 2013; Ryan et al., 2009; Scott, 2002).

Using data on the job preferences of health workers in Ethiopia, our findings add to this nascent body of literature and show that respondents don't always comply with the axiom of continuous preferences in DCEs. Moreover, our analysis also underlines that ANA may sometimes be confused with the low valuation of attributes, although the latter provides valid information about respondents' preferences. We demonstrate that the ANA‐MMNL, which accounts for preference heterogeneity, outperforms the ANA‐LC in terms of goodness of fit. The estimated ANA probabilities are substantially lower in the ANA‐MMNL than in the ANA‐LC, which may imply that health workers with weaker preferences were wrongly classified as non‐attenders in the simpler model. Non‐attendance is noticeable in the more flexible ANA‐MMNL models as well, so its' not the case that accounting for random heterogeneity in preferences will get rid of non‐attendance all together. Rather, allowing for both ANA and preference heterogeneity simultaneously, provides a better picture of respondents' decision‐making behavior than either the ANA‐LC or the MMNL. We also find substantial variation in the rates of ANA exhibited by different health worker cadres. It was noticeable that non‐patient facing staff showed statistically significant ANA for more number of attributes, in comparison to HEWs and mid‐level providers. This was not surprising as HEWs and mid‐level healthcare providers are more used to making choices similar to those in the experiment (such as choosing between different medical treatments) on a regular basis and so the prevalence of simplifying shortcuts was less common in these groups in comparison to health facility administrators (comprising non‐patient facing staff). Our findings were in line with those from Hole et al. (2013), who also demonstrated the use of these models on data from a DCE on doctors' choice of medication, using similar specifications.

The methods in our paper were subject to a number of limitations. Firstly, there has been an ongoing debate about how many draws one should use to make the results of simulation based models of “satisfying” quality. While the debate continues, for the MMNL and ANA‐MMNL models in our paper, we decided to use 5000 Sobol draws which was substantially higher than those used in previous studies in similar contexts (Hess et al., 2013; Hole et al., 2013). Using more draws is always better then using fewer because not only do the estimates become more precise due to reduced simulation error (Czajkowski & Budziński, 2019), a higher number of draws also helps in uncovering any identification problems (Chiou & Walker, 2007). Our choice and number of draws was further guided by the results of Czajkowski et al., who showed that using over 2000 Sobol draws in the case of a DCE with five attributes could be enough to reach sufficient simulation precision. Further, we believe that the lack of a qualitative approach for the selection of attributes in our paper could have been a limitation. Its' crucial to make sure that the chosen attributes and levels are salient to respondents, as no experimental design or econometric analysis can compensate for wrongly defined attributes (Coast et al., 2012). We do strongly believe that our method for selecting attributes was reasonable and the results from our pilot confirmed that respondents had a good understanding of the choice tasks. The format of the choice tasks and the way they are administered can also urge respondents to adopt heuristics in DCEs. To mitigate the possibility of respondents ignoring attributes due to the format of our choice tasks, we chose a design that was similar to and well grounded in recent literature on health workforce DCEs (Mandeville et al., 2016; Saran et al., 2020; Takemura et al., 2016). As a token of our appreciation for the respondent's time, we provided to them a small amount of mobile credit. Since the DCE was administered using a tablet, hand held by the respondent themselves and not overseen by research assistants, we think the chances of social desirability bias or “strategic answering” were also minimal. There is some debate on the use of text versus images to represent the attributes and levels. We opted to display choice tasks only as text since pictures can convey their own meanings, sometimes different from the text, which can misrepresent the attribute levels (Veldwijk et al., 2015). Due to our decision to include salary as a qualitative attribute, we were unable to include willingness‐to‐pay estimates in the study which could have provided useful welfare estimates.

A surprising result was that the coefficient associated with the average salary level was negative, implying that both HEWs and Other cadres preferred a lower‐than‐average salary over an average one. We believe that this result might be due to some misunderstanding of what “average earnings” meant and their corresponding actual value might have been better to include. Respondents may have read quickly and when they saw “20%” they assumed it was “20% higher than average”, not distinguishing between 20% higher and 20% lower. This would even be suggested by the results as there is no statistical difference between above‐average and below‐average (the omitted category) salaries. These results were similar to those of Lamba et al. (2021) who showed that HEWs and non‐patient facing staff did not significantly value higher than average salaries. Without additional research and in the absence of qualitative evidence, however, it is not possible to know whether the validity of these parameter estimates is undermined. Despite the unusual results around the salary attribute, we believe that our study and analysis reflect adequately the preferences expressed by health workers. Our findings were in line with previous health workforce DCEs which report that community level workers often have higher preferences for non‐financial attributes, in comparison to financial remuneration (Abdel‐All et al., 2019; Mandeville et al., 2016; Saran et al., 2020). A study on community health workers from India, for example, demonstrated that more than 85% of the respondents were willing to sacrifice a large proportion of their monthly salary for a job that offered them career progression (Abdel‐All et al., 2019).

Finally, our findings show that while health workers preferred 5 days of training, they had disutility attached to undertaking 10 days of training, compared to no training. We believe that this is a plausible finding as our qualitative research with the sample showed that they did in fact prefer a short training regime, compared to a longer one, as that is less disruptive to their work and doesn't require as much time to catch up with their tasks on their return.

The quantitative analysis of information processing strategies such as ANA is a growing ﬁeld of research in health economics. In particular, studies comparing willingness to pay estimates under the assumption that ANA is a heuristic and ANA is a preference show that its important to disentangle the two to improve policy advice coming from DCEs. For example, wrong assumptions about ANA can effect the estimated benefits and consequently the cost‐benefit ratio in economic evaluations (Heidenreich et al., 2018).

This paper suggests avenues of future research for health economists involved in the study of heuristics in DCEs. First, studying attribute level non‐attendance, instead of just ANA, could lead to further gains in model fit and improve choice predictions. Erdem et al. (2015) demonstrate that in cases where attribute levels are “nominal” (i.e., with no natural sense of ordering), which is common practice in health‐related DCEs, it is possible to study whether respondents, while attending to the attribute as a whole, tend to ignore a subset of attribute levels. We do not explore this in this paper as none of the attributes in our dataset were nominal. Further, in the transport literature for example, it has been reported that respondents sometimes employ a heuristic called “aggregation of common‐metric attributes” where they treat two or more attributes as being identical and simply add them up (Hole et al., 2013). While this was less relevant in this application, since our attributes were qualitative and less amenable to aggregation, it would be useful to study the affects of such heuristics on welfare measures. Finally, it would also be valuable to better understand the motives of respondents for ignoring attributes. For example, in one study respondents ignored the cost attribute to signify their refusal to trade between money and other valued goods such as the environment (Carlsson et al., 2010). Further qualitative research on this topic may be valuable to tease out reasons for non‐attendance in DCEs.

## CONFLICT OF INTEREST

Nikita Arora, Kara Hanson, Mylene Lagarde, Dorka Woldesenbet, Abiy Seifu, Romain Crastes dit Sourd have no conflict of interest to declare. Matthew Quaife holds grants from Bill and Melinda Gates Foundation, outside the submitted work.

## Supporting information

Supplementary Material S1

## Data Availability

Data are available on reasonable request made to the corresponding author.
