# Supplementary material for: Discrete choice analysis of health worker job preferences in Ethiopia: Separating attribute non‐attendance from taste heterogeneity
Source: Health Econ. 2022 Feb 17;31(5):806–19. doi: 10.1002/hec.4475 (PMC9305885; doi:10.1002/hec.4475)
Supplement: Supplementary file 1 — Supplementary Material S1 [file HEC-31-806-s001.pdf]

ONLINE SUPPLEMENTARY INFORMATION

THE ATTRIBUTE NON-ATTENDANCE LATENT CLASS MODEL (ANA-LC)

First we start by providing information on how an attribute non-attendance latent class model (ANA-LC) differs from a standard LCM which is specified to estimate heterogeneity in respondent preferences. We also provide details on the specification of the 64 class ANA-LC used in our analysis, focusing on the issue around identification of parameters for each one of the classes, which can affect model parsimony. Finally, we provide average class attendance for each class/ANA combination in the Results section.

Latent class models for preference heterogeneity and ANA-LC

In a latent class model (LCM) modelling preference heterogeneity, marginal utilities are estimated for each group or class in the population. For example, in a two class LCM with two attributes, the utility function for individual n, alternative j at time t of each of the two classes can be written as follows:

$$u_{njt}^{class1} = \beta_1^{class1} attribute1_{njt} + \beta_2^{class1} attribute1_{njt} + \varepsilon_{njt}$$

$$u_{njt}^{class2} = \beta_1^{class2} attribute2_{njt} + \beta_2^{class2} attribute2_{njt} + \varepsilon_{njt}$$

When specifying the above model, four different marginal utilities and thus four different parameters would need to be identified (β_1^{class1},β_2^{class1},β_1^{class2},β_2^{class2}). With a rise in the number of classes in a model, the number of parameters that need to be identified also quickly rise. The way an LCM that's modelling heterogeneity in respondent preferences is specified, is often different from an ANA-LC. In the former, the number of latent classes in which respondents could be allocated to is unknown to the analyst a priori. The optimal number of classes is normally determined by noticing the change in model goodness of fit as the number of classes go up one-by-one. This can be done by monitoring an information criterion like AIC or BIC which penalizes model fit as the number of parameters increase.

In contrast, an ANA-LC estimates a behavioral model which assumes that respondents use heuristics in processing information in a DCE, and only attend to a subset of the given K attributes. This results in 2^K different combinations of ANA. In this model, the number of classes in which the respondents could be allocated to coincides with the combinations with which ANA could occur and is known to the analyst beforehand (Heidenreich et al., 2018). Scarpa et al (Scarpa et al., 2009) and Collins et al (Collins, 2012) have demonstrated that researchers can end up with misleading estimates of ANA shares if a reduced version of the ANA model with less classes is estimated. This could be because the classes may not be independent.

Therefore, we estimated an ANA model with all 2^K classes. Researchers in Health Economics have previously used a step-wise approach to modeling all possible combinations of ANA (Lagarde, 2013), including only those classes in their ANA-LCs, that that are nonempty. Whether a class ends up being empty or not is dependent on the value of the corresponding constant in a class allocation model. Researchers generally estimate a multinomial logit model with one constant per class, one of which is the base and is thus constrained to be zero.

An alternative to this approach is to estimate a model with 2^K classes but use a different parameterization for the LCM where class allocation probabilities are not captured by a (large) set of constants, but by a product of logit probabilities corresponding to the probability of each attribute being attended to or not (Hole et al., 2013). This allows to reduce the number of parameters estimated, maintaining model parsimony. In the context of this paper, this means that we consider 64 classes corresponding to different patterns of ANA, but this only requires the estimation of 6 additional parameters instead of 63 (64 minus 1).

METHOD

As explained above, we estimated a constant for each of the six attributes and generated the probability of an attribute being attended to, over all 64 combinations, by introducing a binary logit model for each of the attributes, which was specified as:

$$P_{attribute} = \delta_{attribute} + HEW_{attribute} * HEW + patientfacing_{attribute} * patientfacing \ [1]$$

Where $\delta_{attribute}$ was the constant for a given attribute; $HEW_{attribute}$ and $patientfacing_{attribute}$ were constants for that attribute, each for the corresponding covariate of two cadres – HEWs (health extension workers) and patient-facing staff.

For example, the way we specified the probability of respondents attending to Salary was given by

$$P_{salary} = \delta_{salary} + HEW_{salary} * HEW + patientfacing_{salary} * patientfacing \ [2]$$

Each of the 64 combinations of ANA were then specified as a separate class using equation 1, where when an attribute was considered to have been ignored, it was restricted to zero. For example, the probability of a combination where all attributes were attended to except for *salary* and *workload,* corresponded to:

$$\omega_{salary\ and\ workload\ non-attendance} =$$

$$\frac{1}{1+\exp(\delta_{salary})} \cdot \frac{\exp(\delta_{training})}{1+\exp(\delta_{training})} \cdot \frac{1}{1+\exp(\delta_{Workload})} \cdot \frac{\exp(\delta_{quality})}{1+\exp(\delta_{quality})} \cdot \frac{\exp(\delta_{management})}{1+\exp(\delta_{management})} \cdot \frac{\exp\ (\delta_{opportunities})}{1+\exp\ (\delta_{opportunities})} [$$

And was specified as,

$$P\ (class_1) = \ 0 + P_{training} + 0 + P_{quality} + P_{management} + P_{opportunities}$$

RESULTS

Table 1 describes the patterns of non-attendance represented in each class and the proportion of HEWs who adopted those strategies. Table 2 describes the same but for Other cadres. Where an attribute was considered to be ignored, the parameter weight was restricted to ‘0’ in the model.

Table 1: Average class membership of HEWs in the ANA-LC

		ANA patterns tested								
**Class number**	**Average class membership**	**Salary offered**		**Training offered**		**Workload**		**Good facility quality**	**Good management**	**Good outcome**
Class_1	0.53%	0	0	0	0	β_workmed	β_workheav	β_gdfacqual	β_gdmgmt	β_gdoutcome
Class_2	2.84%	0	0	β_train5	β_train10	0	0	β_gdfacqual	β_gdmgmt	β_gdoutcome
Class_3	1.38%	0	0	β_train5	β_train10	β_workmed	β_workheav	0	β_gdmgmt	β_gdoutcome
Class_4	0.16%	0	0	β_train5	β_train10	β_workmed	β_workheav	β_gdfacqual	0	β_gdoutcome
Class_5	1.53%	0	0	β_train5	β_train10	β_workmed	β_workheav	β_gdfacqual	β_gdmgmt	0
Class_6	0.00%	β_salavg	β_salplus	0	0	0	0	β_gdfacqual	β_gdmgmt	β_gdoutcome
Class_7	0.00%	β_salavg	β_salplus	0	0	β_workmed	β_workheav	0	β_gdmgmt	β_gdoutcome
Class_8	0.00%	β_salavg	β_salplus	0	0	β_workmed	β_workheav	β_gdfacqual	0	β_gdoutcome
Class_9	0.00%	β_salavg	β_salplus	0	0	β_workmed	β_workheav	β_gdfacqual	β_gdmgmt	0
Class_10	0.00%	β_salavg	β_salplus	β_train5	β_train10	0	0	0	β_gdmgmt	β_gdoutcome
Class_11	0.00%	β_salavg	β_salplus	β_train5	β_train10	0	0	β_gdfacqual	0	β_gdoutcome
Class_12	0.00%	β_salavg	β_salplus	β_train5	β_train10	0	0	β_gdfacqual	β_gdmgmt	0
Class_13	0.00%	β_salavg	β_salplus	β_train5	β_train10	β_workmed	β_workheav	0	0	β_gdoutcome
Class_14	0.00%	β_salavg	β_salplus	β_train5	β_train10	β_workmed	β_workheav	0	β_gdmgmt	0
Class_15	0.00%	β_salavg	β_salplus	β_train5	β_train10	β_workmed	β_workheav	β_gdfacqual	0	0
Class_16	2.57%	0	0	0	0	0	0	β_gdfacqual	β_gdmgmt	β_gdoutcome
Class_17	1.25%	0	0	0	0	β_workmed	β_workheav	0	β_gdmgmt	β_gdoutcome
Class_18	0.15%	0	0	0	0	β_workmed	β_workheav	β_gdfacqual	0	β_gdoutcome
Class_19	1.38%	0	0	0	0	β_workmed	β_workheav	β_gdfacqual	β_gdmgmt	0
Class_20	6.64%	0	0	β_train5	β_train10	0	0	0	β_gdmgmt	β_gdoutcome
Class_21	0.78%	0	0	β_train5	β_train10	0	0	β_gdfacqual	0	β_gdoutcome
Class_22	7.36%	0	0	β_train5	β_train10	0	0	β_gdfacqual	β_gdmgmt	0

Class_23	0.38%	0	0	β_train5	β_train10	β_workmed	β_workheav	0	0	β_gdoutcome
Class_24	3.58%	0	0	β_train5	β_train10	β_workmed	β_workheav	0	β_gdmgmt	0
Class_25	0.42%	0	0	β_train5	β_train10	β_workmed	β_workheav	β_gdfacqual	0	0
Class_26	0.00%	β_salavg	β_salplus	0	0	0	0	0	β_gdmgmt	β_gdoutcome
Class_27	0.00%	β_salavg	β_salplus	0	0	0	0	β_gdfacqual	0	β_gdoutcome
Class_28	0.00%	β_salavg	β_salplus	0	0	0	0	β_gdfacqual	β_gdmgmt	0
Class_29	0.00%	β_salavg	β_salplus	0	0	β_workmed	β_workheav	0	0	β_gdoutcome
Class_30	0.00%	β_salavg	β_salplus	0	0	β_workmed	β_workheav	0	β_gdmgmt	0
Class_31	0.00%	β_salavg	β_salplus	0	0	β_workmed	β_workheav	β_gdfacqual	0	0
Class_32	0.00%	β_salavg	β_salplus	β_train5	β_train10	0	0	0	0	β_gdoutcome
Class_33	0.00%	β_salavg	β_salplus	β_train5	β_train10	0	0	0	β_gdmgmt	0
Class_34	0.00%	β_salavg	β_salplus	β_train5	β_train10	0	0	β_gdfacqual	0	0
Class_35	0.00%	β_salavg	β_salplus	β_train5	β_train10	β_workmed	β_workheav	0	0	0
Class_36	6.02%	0	0	0	0	0	0	0	β_gdmgmt	β_gdoutcome
Class_37	0.71%	0	0	0	0	0	0	β_gdfacqual	0	β_gdoutcome
Class_38	6.66%	0	0	0	0	0	0	β_gdfacqual	β_gdmgmt	0
Class_39	0.34%	0	0	0	0	β_workmed	β_workheav	0	0	β_gdoutcome
Class_40	3.24%	0	0	0	0	β_workmed	β_workheav	0	β_gdmgmt	0
Class_41	0.38%	0	0	0	0	β_workmed	β_workheav	β_gdfacqual	0	0
Class_42	1.83%	0	0	β_train5	β_train10	0	0	0	0	β_gdoutcome
Class_43	17.23%	0	0	β_train5	β_train10	0	0	0	β_gdmgmt	0
Class_44	2.03%	0	0	β_train5	β_train10	0	0	β_gdfacqual	0	0
Class_45	0.99%	0	0	β_train5	β_train10	β_workmed	β_workheav	0	0	0
Class_46	0.00%	β_salavg	β_salplus	0	0	0	0	0	0	β_gdoutcome
Class_47	0.00%	β_salavg	β_salplus	0	0	0	0	0	β_gdmgmt	0
Class_48	0.00%	β_salavg	β_salplus	0	0	0	0	β_gdfacqual	0	0
Class_49	0.00%	β_salavg	β_salplus	0	0	β_workmed	β_workheav	0	0	0
Class_50	0.00%	β_salavg	β_salplus	β_train5	β_train10	0	0	0	0	0

Class_51	1.66%	0	0	0	0	0	0	0	0	β_gdoutcome
Class_52	15.60%	0	0	0	0	0	0	0	β_gdmgmt	0
Class_53	1.84%	0	0	0	0	0	0	β_gdfacqual	0	0
Class_54	0.89%	0	0	0	0	β_workmed	β_workheav	0	0	0
Class_55	4.75%	0	0	β_train5	β_train10	0	0	0	0	0
Class_56	0.00%	β_salavg	β_salplus	0	0	0	0	0	0	0
Class_57	0.59%	0	0	β_train5	β_train10	β_workmed	β_workheav	β_gdfacqual	β_gdmgmt	β_gdoutcome
Class_58	0.00%	β_salavg	β_salplus	0	0	β_workmed	β_workheav	β_gdfacqual	β_gdmgmt	β_gdoutcome
Class_59	0.00%	β_salavg	β_salplus	β_train5	β_train10	0	0	β_gdfacqual	β_gdmgmt	β_gdoutcome
Class_60	0.00%	β_salavg	β_salplus	β_train5	β_train10	β_workmed	β_workheav	0	β_gdmgmt	β_gdoutcome
Class_61	0.00%	β_salavg	β_salplus	β_train5	β_train10	β_workmed	β_workheav	β_gdfacqual	0	β_gdoutcome
Class_62	0.00%	β_salavg	β_salplus	β_train5	β_train10	β_workmed	β_workheav	β_gdfacqual	β_gdmgmt	0
Class_63	4.30%	0	0	0	0	0	0	0	0	0
Class_64	0.00%	β_salavg	β_salplus	β_train5	β_train10	β_workmed	β_workheav	β_gdfacqual	β_gdmgmt	β_gdoutcome

Table 2: Average class membership of Other cadres in the ANA-LC

		**ANA patterns tested**								
**Class number**	**Average class membership**	**Salary offered**		**Training offered**		**Workload**		**Good facility quality**	**Good management**	**Good outcome**
Class_1	0.22%	0	0	0	0	β_workmed	β_workheav	β_gdfacqual	β_gdmgmt	β_gdoutcome
Class_2	0.12%	0	0	β_train5	β_train10	0	0	β_gdfacqual	β_gdmgmt	β_gdoutcome
Class_3	0.19%	0	0	β_train5	β_train10	β_workmed	β_workheav	0	β_gdmgmt	β_gdoutcome
Class_4	0.12%	0	0	β_train5	β_train10	β_workmed	β_workheav	β_gdfacqual	0	β_gdoutcome

Class_5	0.03%	0	0	β_train5	β_train10	β_workmed	β_workheav	β_gdfacqual	β_gdmgmt	0
Class_6	1.16%	β_salavg	β_salplus	0	0	0	0	β_gdfacqual	β_gdmgmt	β_gdoutcome
Class_7	1.87%	β_salavg	β_salplus	0	0	β_workmed	β_workheav	0	β_gdmgmt	β_gdoutcome
Class_8	0.89%	β_salavg	β_salplus	0	0	β_workmed	β_workheav	β_gdfacqual	0	β_gdoutcome
Class_9	0.28%	β_salavg	β_salplus	0	0	β_workmed	β_workheav	β_gdfacqual	β_gdmgmt	0
Class_10	1.00%	β_salavg	β_salplus	β_train5	β_train10	0	0	0	β_gdmgmt	β_gdoutcome
Class_11	0.45%	β_salavg	β_salplus	β_train5	β_train10	0	0	β_gdfacqual	0	β_gdoutcome
Class_12	0.15%	β_salavg	β_salplus	β_train5	β_train10	0	0	β_gdfacqual	β_gdmgmt	0
Class_13	0.73%	β_salavg	β_salplus	β_train5	β_train10	β_workmed	β_workheav	0	0	β_gdoutcome
Class_14	0.24%	β_salavg	β_salplus	β_train5	β_train10	β_workmed	β_workheav	0	β_gdmgmt	0
Class_15	0.11%	β_salavg	β_salplus	β_train5	β_train10	β_workmed	β_workheav	β_gdfacqual	0	0
Class_16	0.63%	0	0	0	0	0	0	β_gdfacqual	β_gdmgmt	β_gdoutcome
Class_17	1.02%	0	0	0	0	β_workmed	β_workheav	0	β_gdmgmt	β_gdoutcome
Class_18	1.08%	0	0	0	0	β_workmed	β_workheav	β_gdfacqual	0	β_gdoutcome
Class_19	0.15%	0	0	0	0	β_workmed	β_workheav	β_gdfacqual	β_gdmgmt	0
Class_20	0.53%	0	0	β_train5	β_train10	0	0	0	β_gdmgmt	β_gdoutcome
Class_21	0.32%	0	0	β_train5	β_train10	0	0	β_gdfacqual	0	β_gdoutcome

Class_22	0.08%	0	0	β_train5	β_train10	0	0	β_gdfacqual	β_gdmgmt	0
Class_23	0.53%	0	0	β_train5	β_train10	β_workmed	β_workheav	0	0	β_gdoutcome
Class_24	0.13%	0	0	β_train5	β_train10	β_workmed	β_workheav	0	β_gdmgmt	0
Class_25	0.08%	0	0	β_train5	β_train10	β_workmed	β_workheav	β_gdfacqual	0	0
Class_26	5.28%	β_salavg	β_salplus	0	0	0	0	0	β_gdmgmt	β_gdoutcome
Class_27	2.48%	β_salavg	β_salplus	0	0	0	0	β_gdfacqual	0	β_gdoutcome
Class_28	0.79%	β_salavg	β_salplus	0	0	0	0	β_gdfacqual	β_gdmgmt	0
Class_29	4.02%	β_salavg	β_salplus	0	0	β_workmed	β_workheav	0	0	β_gdoutcome
Class_30	1.27%	β_salavg	β_salplus	0	0	β_workmed	β_workheav	0	β_gdmgmt	0
Class_31	0.59%	β_salavg	β_salplus	0	0	β_workmed	β_workheav	β_gdfacqual	0	0
Class_32	2.05%	β_salavg	β_salplus	β_train5	β_train10	0	0	0	0	β_gdoutcome
Class_33	0.68%	β_salavg	β_salplus	β_train5	β_train10	0	0	0	β_gdmgmt	0
Class_34	0.30%	β_salavg	β_salplus	β_train5	β_train10	0	0	β_gdfacqual	0	0
Class_35	0.49%	β_salavg	β_salplus	β_train5	β_train10	β_workmed	β_workheav	0	0	0
Class_36	2.86%	0	0	0	0	0	0	0	β_gdmgmt	β_gdoutcome
Class_37	2.78%	0	0	0	0	0	0	β_gdfacqual	0	β_gdoutcome
Class_38	0.42%	0	0	0	0	0	0	β_gdfacqual	β_gdmgmt	0

Class_39	4.65%	0	0	0	0	β_workmed	β_workheav	0	0	β_gdoutcome
Class_40	0.69%	0	0	0	0	β_workmed	β_workheav	0	β_gdmgmt	0
Class_41	0.62%	0	0	0	0	β_workmed	β_workheav	β_gdfacqual	0	0
Class_42	1.43%	0	0	β_train5	β_train10	0	0	0	0	β_gdoutcome
Class_43	0.36%	0	0	β_train5	β_train10	0	0	0	β_gdmgmt	0
Class_44	0.20%	0	0	β_train5	β_train10	0	0	β_gdfacqual	0	0
Class_45	0.33%	0	0	β_train5	β_train10	β_workmed	β_workheav	0	0	0
Class_46	11.23%	β_salavg	β_salplus	0	0	0	0	0	0	β_gdoutcome
Class_47	3.58%	β_salavg	β_salplus	0	0	0	0	0	β_gdmgmt	0
Class_48	1.66%	β_salavg	β_salplus	0	0	0	0	β_gdfacqual	0	0
Class_49	2.68%	β_salavg	β_salplus	0	0	β_workmed	β_workheav	0	0	0
Class_50	1.38%	β_salavg	β_salplus	β_train5	β_train10	0	0	0	0	0
Class_51	12.01%	0	0	0	0	0	0	0	0	β_gdoutcome
Class_52	1.93%	0	0	0	0	0	0	0	β_gdmgmt	0
Class_53	1.62%	0	0	0	0	0	0	β_gdfacqual	0	0
Class_54	2.69%	0	0	0	0	β_workmed	β_workheav	0	0	0
Class_55	0.91%	0	0	β_train5	β_train10	0	0	0	0	0
Class_56	7.51%	β_salavg	β_salplus	0	0	0	0	0	0	0

Class_57	0.04%	0	0	β_train5	β_train10	β_workmed	β_workheav	β_gdfacqual	β_gdmgmt	β_gdoutcome
Class_58	0.41%	β_salavg	β_salplus	0	0	β_workmed	β_workheav	β_gdfacqual	β_gdmgmt	β_gdoutcome
Class_59	0.22%	β_salavg	β_salplus	β_train5	β_train10	0	0	β_gdfacqual	β_gdmgmt	β_gdoutcome
Class_60	0.35%	β_salavg	β_salplus	β_train5	β_train10	β_workmed	β_workheav	0	β_gdmgmt	β_gdoutcome
Class_61	0.16%	β_salavg	β_salplus	β_train5	β_train10	β_workmed	β_workheav	β_gdfacqual	0	β_gdoutcome
Class_62	0.05%	β_salavg	β_salplus	β_train5	β_train10	β_workmed	β_workheav	β_gdfacqual	β_gdmgmt	0
Class_63	7.04%	0	0	0	0	0	0	0	0	0
Class_64	0.08%	β_salavg	β_salplus	β_train5	β_train10	β_workmed	β_workheav	β_gdfacqual	β_gdmgmt	β_gdoutcome

REFERENCES

Collins, A. (2012). Attribute nonattendance in discrete choice models: measurement of bias, and a model for the inference of both nonattendance and taste heterogeneity.

Heidenreich, S., Watson, V., Ryan, M., & Phimister, E. (2018). Decision heuristic or preference? Attribute non-attendance in discrete choice problems. *Health economics,* 27, 157-171.

Hole, A.R., Kolstad, J.R., & Gyrd-Hansen, D. (2013). Inferred vs. stated attribute non-attendance in choice experiments: A study of doctors' prescription behaviour. *Journal of Economic Behavior & Organization,* 96, 21-31.

Lagarde, M. (2013). Investigating attribute non-attendance and its consequences in choice experiments with latent class models. *Health economics,* 22, 554-567.

Scarpa, R., Gilbride, T.J., Campbell, D., & Hensher, D.A. (2009). Modelling attribute non-attendance in choice experiments for rural landscape valuation. *European review of agricultural economics,* 36, 151-174.
